# Secreted and tumour targeted human carboxylesterase for activation of irinotecan

**DOI:** 10.1038/sj.bjc.6600519

**Published:** 2002-09-04

**Authors:** D Oosterhoff, H M Pinedo, I H van der Meulen, M de Graaf, T Sone, F A Kruyt, V W van Beusechem, H J Haisma, W R Gerritsen

**Affiliations:** Division of Gene Therapy, Department of Medical Oncology, Vrije Universiteit Medical Center, PO Box 7057, 1007 MB, Amsterdam, The Netherlands; Department of Toxicology, Faculty of Pharmaceutical Sciences, Setsunan University, Osaka, Japan; Department of Therapeutic Gene Modulation, University Center for Pharmacy, PO Box 196, 9700 AD, Groningen, The Netherlands

**Keywords:** cancer, carboxylesterase, chemotherapy, CPT-11, prodrug, SN-38

## Abstract

Irinotecan (CPT-11) is an anticancer agent for the treatment of colon cancer. CPT-11 can be considered as a prodrug, since it needs to be activated into the toxic drug SN-38 by the enzyme carboxylesterase. An approach to achieve tumour specific activation of CPT-11 is to transduce the cDNA encoding carboxylesterase into tumour cells. A secreted form of carboxylesterase may diffuse through a tumour mass and may activate CPT-11 extracellularly. This could enhance the antitumour efficacy by exerting a bystander effect on untransduced cells. In addition a secreted tumour-targeted form of carboxylesterase should prevent leakage of the enzyme from the site of the tumour into the circulation. We have constructed a secreted form of human liver carboxylesterase-2 by deletion of the cellular retention signal and by cloning the cDNA downstream of an Ig kappa leader sequence. The protein was secreted by transfected cells and showed both enzyme activity and efficient CPT-11 activation. To obtain a secreted, tumour-targeted form of carboxylesterase-2 the cDNA encoding the human scFv antibody C28 directed against the epithelial cell adhesion molecule EpCAM, was inserted between the leader sequence and carboxylesterase-2. This fusion protein showed CPT-11 activation and specific binding to EpCAM expressing cells. Importantly, in combination with CPT-11 both recombinant carboxylesterase proteins exerted strong antiproliferative effects on human colon cancer cells. They are, therefore, promising new tools for gene directed enzyme prodrug therapy approaches for the treatment of colon carcinoma with CPT-11.

*British Journal of Cancer* (2002) **87**, 659–664. doi:10.1038/sj.bjc.6600519 www.bjcancer.com

© 2002 Cancer Research UK

## 

Conventional chemotherapy lacks specificity for tumour cells. This results in dose-limiting side effects and insufficient concentrations of the drugs in the tumour, through which efficacy is limited and drug resistant cellular subpopulations may emerge. These problems may be overcome by expressing an enzyme that is capable of converting a non-toxic prodrug into a toxic drug specifically in tumour cells. This so-called gene-directed enzyme prodrug therapy (GDEPT) or suicide gene therapy aims to increase the concentration of the drug in the tumour while reducing the systemic toxicity. The gene encoding the prodrug-activating enzyme is delivered to the tumour cells by, for example, an adenoviral vector, followed by systemic administration of the prodrug. In this regard, several prodrug-converting enzymes have been extensively studied, such as the herpes simplex virus thymidine kinase enzyme that converts ganciclovir (GCV) into the active compound GCV-P and bacterial cytosine deaminase that activates 5-FC to the anticancer drug 5-FU (Moolten, 1986; Austin and Huber, 1993).

A prodrug for the treatment of colon carcinoma is irinotecan (CPT-11 or 7-ethyl-10-[4-(1-piperidino)-1-piperidino] carbonyloxycamptothecin). CPT-11 is converted by carboxylesterases (CE) into the toxic drug SN-38 (7-ethyl-10-hydroxycamptothecin) by cleavage of the bulky dipiperidino side chain at the carbon position (Tsuji *et al*, 1991; Satoh *et al*, 1994). CPT-11 has demonstrated antitumour activity in immune deprived animals bearing human tumour xenografts (Houghton *et al*, 1995, 1996; Thompson *et al*, 1997a,b) and is approved for use in the treatment of metastatic colorectal cancer in humans. Although SN-38 can be detected in the plasma of cancer patients only minutes after the administration of CPT-11 (Gupta *et al*, 1997), 90% of the administered CPT-11 is not converted to SN-38 (Rivory *et al*, 1997).

CEs are a ubiquitously expressed class of enzymes. High levels of enzyme activity are found in human liver and lung (Satoh and Hosokawa, 1998). Different isoforms of human CE have been described. CE1 is found in liver only, whereas CE2 is also found in the intestines and CE3 is found in brain cells (Hojring and Svensmark, 1977; Ketterman *et al*, 1989). Furthermore, it has been shown that human alveolar macrophages release a serine esterase that is identical to liver CE1 (Munger *et al*, 1991).

Several studies have been performed using CPT-11 in combination with human CE1 in a GDEPT approach. Kojima *et al* (1998a,b) described the construction of a replication deficient adenoviral vector containing the human liver CE1 gene driven by the CMV promoter. *In vitro* results showed that several tumour cell lines infected with this virus express CE1 and in the presence of CPT-11 tumour growth was effectively suppressed. However, on many other tumour cell lines only minimal effects were observed. This underscored the notice that the success of a GDEPT approach for CPT-11 requires an enzyme with a high efficiency of converting CPT-11 to SN-38. The rabbit CE was found to be 100–1000-fold more efficient in converting CPT-11 than human liver CE1 and was 12–55-fold more efficient in sensitising transfected cells to CPT-11 (Danks *et al*, 1999). Therefore, an adenoviral vector expressing rabbit CE was constructed and transduction of human tumour cells led to sensitisation to CPT-11 (Wierdl *et al*, 2001). The disadvantage of rabbit CE, however, is that expression of a nonhuman protein in patients may lead to an immunological response and subsequent enzyme inactivation. A human enzyme with higher affinity and higher efficiency than CE1 may overcome these limitations. It was shown that human CE2 has a higher affinity and a higher conversion velocity for CPT-11 than CE1 (Humerickhouse *et al*, 2000). Therefore, we envisaged that CE2 would be a candidate to employ in a GDEPT approach to treat human tumours.

To achieve efficient kill of all tumour cells, a bystander effect is required, whereby CPT-11 is cleaved to SN-38 that not only kills the tumour cells in which CE2 is formed, but also neighbouring tumour cells that do not express CE2. We hypothesised that extracellular conversion of CPT-11 would lead to a larger bystander effect than intracellular conversion and, furthermore, that a fusion protein consisting of secreted CE2 fused to a tumour specific scFv antibody will be retained in the tumour thereby preventing leakage of the enzyme into the circulation and therefore further reducing unwanted side effects.

In this study we describe the construction of a secreted form of CE2 (sCE2) by deletion of a C-terminal cellular retention signal and by adding the Ig kappa leader sequence. Furthermore, a secreted targeted form of human CE2 (C28-sCE2) was constructed by fusing sCE2 to a human scFv directed against Epithelial Cell Adhesion Molecule (EpCAM), a tumour-associated antigen. The binding specificity and enzyme activity of the secreted form of CE2 and the fusion protein and their ability to sensitise human tumour cell lines to CPT-11 is determined and compared to wildtype intracellularly expressed human CE2 (CE2).

## MATERIALS AND METHODS

### Chemicals

Pwo polymerase, PCR buffer and dNTPs were obtained from Roche (Almere, The Netherlands). Restriction enzymes were purchased from New England Biolabs (Beverly, MA, USA) and Life Technologies (Breda, The Netherlands). The kits used for DNA isolation, purification and extraction from agarose gel were from Qiagen (Hilden, Germany). The substrate p-nitrophenyl-acetate was purchased from Sigma-Aldrich (Zwijndrecht, The Netherlands). The prodrug CPT-11 and the drug SN-38 were obtained from Rhône-Poulenc Rorer (Vitry-sur-Seine, France).

### Cell lines

The COS-7 and the human colon cancer SW1398 cell lines were cultured in Dulbecco's modified Eagle's medium (DMEM, Life Technologies, Paisley, UK) supplemented with 5% (COS-7) or 10% heat-inactivated foetal calf serum (Life Technologies), 50 IU ml^−1^ penicillin (Life Technologies) and 50 μg ml^−1^ streptomycin (Life Technologies) in a humidified atmosphere containing 5% CO_2_ at 37°C.

### Construction of psCE2 and pC28-sCE2

**Figure 1:**
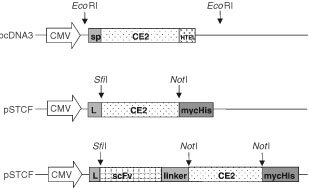
Schematic representation of the CE2, sCE2 and C28-sCE2 expression cassette. The CE2 cDNA is inserted as an *Eco*RI fragment into pcDNA3. The encoded protein contains its wildtype N-terminal signal peptide and a C-terminal cellular retention signal sequence HTEL. The structural elements of pSTCF include the strong cytomegalovirus (CMV) promoter, IgG kappa leader sequence, and a C-terminal myc- and His-tag (mycHis) for easy detection and purification. sCE2, without retention signal, is inserted as a *Sfi*I/*Not*I fragment into pSTCF. The anti-EpCAM scFv C28 is inserted as a *Sfi*I/*Not*I fragment. The gene encoding CE2 is inserted as a *Not*I/*Not*I fragment, after the (Gly_4_Ser)_2_ linker is inserted in the *Not*I and *Apa*I restriction sites.

The pBluescript vector containing the CE2 open reading frame (Sone and Wang, 1997) was digested with *Eco*RI and the CE2 encoding fragment was ligated into the *Eco*RI linearised eukaryotic expression vector pcDNA3 (Invitrogen, Groningen, The Netherlands). This construct was called pCE2. To construct a secreted form of CE2 (psCE2), two primers (sense 5′-GACGC*GGCCCAGCCGGCC*CAGGACTCAGCCAGTCCCATCC-3′ and antisense 5′-GACTCGA*GCGGCCGC*TCTCTCTTCAGGCTCCTCGAGC-3′) were designed to introduce an *Sfi*I restriction site (italic) at the beginning of the sequence encoding the mature protein and a *Not*I restriction site (italic) before the retention signal, as shown in Figure 1

. After performing the PCR, the *Sfi*I/*Not*I digested fragment was isolated, purified, and ligated into the eukaryotic expression vector pSTCF, containing a myc- and 6 his-tag and the Ig kappa leader sequence that directs proteins to the secretory pathway (Arafat *et al*, 2000).

The human anti-EpCAM scFv C28 was derived from scFv UBS-54, which was isolated from a semi-synthetic phage antibody display library (Huls *et al*, 1999), and was a kind gift of Dr T Logtenberg (Crucell, Leiden). An *Sfi*I/*Not*I fragment encoding the scFv C28 was isolated from a pHEN vector, and cloned into the eukaryotic expression vector pSTCF. A flexible (Gly_4_Ser)_2_ linker was introduced downstream of C28, as described previously (de Graaf *et al*, 2002). To allow insertion of sCE2 downstream of the (Gly_4_Ser)_2_ linker, a PCR was performed to obtain a DNA fragment encoding a secreted form of CE2 starting from the mature CE2 protein and ending just before the cellular retention signal. Both primers used in the PCR (sense 5′-GTGT*GCGGCCGC*CAGGACTCAGCCAGTCCCATC-3′ and antisense primer as described above) contained a *Not*I site (italic). The PCR product was digested with *Not*I and inserted into the *Not*I sites of the vector containing C28 with the (Gly_4_Ser)_2_ linker to obtain pC28-sCE2.

### Expression of CE2, sCE2 and C28-sCE2 fusion protein

COS-7 cells (2×10^6^) were transfected with 2 μg pCE2, psCE2 or pC28-sCE2 by Lipofectamine Plus reagent (Life Technologies) according to instructions of the manufacturer. Cells were grown in 3.5 ml DMEM containing 5% FCS and antibiotics. After 48 h, supernatants were removed and cells were harvested by trypsinization. Cellular lysates were obtained by three times freeze thawing in 350 μl PBS. For cytotoxicity assays and HPLC analysis proteins present in supernatants of transfected COS-7 cells were 10× concentrated using a Biomax-10 centrifugal filter (Millipore, Bedford, USA). Supernatants and cellular lysates were analysed for the presence of functional CE enzyme or C28-sCE2 fusion protein by Western blotting, esterase activity assay and cytotoxicity assays. Binding of proteins in supernatants of COS-7 cells transfected with pC28-sCE2 or psCE2 to EpCAM positive cells was determined by FACS analysis.

### Western blot analysis

Proportional amounts of supernatant or cellular lysates from COS-7 cells transfected with pCE2, psCE2 or pC28-sCE2 were dissolved in sample buffer (Laemmli, 1970) with 5% 2-mercaptoethanol and heated at 95°C for 5 min. Samples were electrophoresed through a denaturing 10% sodium dodecyl sulphate-polyacrylamide gel and protein bands were electroblotted onto PVDF protein membrane (BioRad). Proteins were detected using anti-myc antibody 9E10 (Chan *et al*, 1987) and HRP-conjugated rabbit anti-mouse IgG (Dako) or with rabbit-anti-CE2, an antibody directed to the C-terminal retention signal of CE which was a kind gift of Dr Yan, University of Rhode Island (Zhu *et al*, 2000), and HRP-conjugated swine anti-rabbit IgG (Dako). Blots were developed with enhanced chemo luminescence reagent (Lumilight Plus, Roche).

### Esterase activity assay

Supernatants or cellular lysates of transfected COS-7 cells were incubated with 200 μl 100 mM Tris-HCl pH 8.0 containing 100 mM pNpAc, a substrate for CE. After mixing, conversion to p-Nitrophenol was measured at a wavelength of 415 nm during 10 min using an ELISA plate reader (BioRad, Veenendaal, The Netherlands).

### FACS analysis

EpCAM expressing SW1398 cells were trypsinised for 5 min at 37°C, washed with DMEM, counted and resuspended in PBS. A total of 5×10^5^ cells was incubated for 1 h on ice with 50 μl supernatant of COS-7 cells transfected with pC28-sCE2. As a negative control, supernatants of untransfected COS-7 cells or cells transfected with psCE2 were used. After washing three times with PBS, cells were incubated with anti-myc antibody 9E10 in PBS/0.1% BSA, washed three times with PBS, and stained with fluorescein-conjugated rabbit anti-mouse IgG (Dako). As a positive control 50 μl (10 μg ml^−1^) of the anti-EpCAM antibody 323/A3 (Edwards *et al*, 1986) was used. Stained cells were fixed with 1% formaldehyde in PBS and analysed on a FACScan flow cytometer (Becton Dickinson, Mountain View, CA, USA).

### Immunohistochemistry

To show binding of C28-sCE2 to EpCAM expressing cells, 1×10^4^ SW1398 cells were plated and incubated overnight with the concentrated supernatants of COS-7 cells transfected with psCE2 or pC28-sCE2. Unbound enzyme was removed by washing with culture medium and cells were fixed with 100 μl 50% MeOH/50% acetone. After washing with PBS, anti-myc antibody 9E10 was added to the cells for 1 h at 37°C, followed by incubation with rabbit anti mouse HRP (1 : 100 in PBS/0.1%BSA) for 1 h. Hereafter cells were washed and 3-amino-9-ethylcarbazole substrate chromogen (Dako, USA) was added. The staining was stopped by washing with PBS. Cells were counterstained with haematoxylin.

### *In vitro* cytotoxicity assay

SW1398 cells (1×10^4^) were plated in a 96-well microtiter plate (Bio-one). After 24 h, concentrated supernatants of COS-7 cells transfected with pCE2, psCE2 or pC28-sCE2 was added together with a non-toxic concentration of CPT-11 (1 μM). Control experiments were performed in which SW1398 cells were incubated with DMEM supplemented with 10% FCS, SN-38 or CPT-11 only. After another 72 h culture the cells were incubated with cell proliferation reagent WST-1 (Roche Diagnostics) for 1 h at 37°C. The absorbency was measured at a wavelength of 450 nm. The antiproliferative effects were determined and expressed as percentages of growth as compared to untreated control growth, which was set to 100%.

## RESULTS

### Construction of CE2, sCE2 and C28-sCE2

The cDNA coding for human CE2 (Sone and Wang, 1997) was inserted into the eukaryotic expression vector pcDNA3, creating pCE2 (Figure 1). Using PCR we amplified a CE2 cDNA fragment encoding the mature protein without the last four amino acids encoding the cellular retention signal HTEL. This fragment was inserted into the pSTCF vector, which contains the Ig kappa leader that directs the protein in the secretory pathway and a myc- and 6xhis-tag (Arafat *et al*, 2000). The resulting construct encoding a secreted form of CE2 (sCE2) was designated psCE2 (Figure 1). The cDNA fragment coding for sCE2 was also inserted into the pSTCF vector in frame with the anti-EpCAM scFv C28, creating pC28-sCE2 (Figure 1).

### Expression and characterisation of CE2, sCE2 and C28-sCE2

**Figure 2:**
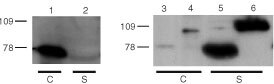
Western blot analysis of the cellular lysates and supernatants of COS-7 cells transfected with pCE, psCE2 or pC28-sCE2. sCE2 and C28-sCE2 were detected using an antibody directed against the myc-tag and CE2 was detected with an antibody directed against the C-terminal cellular retention signal. In lanes 1, 3 and 4 cellular lysates (c) and in lanes 2, 5 and 6 supernatants (s) of COS-7 cells transfected with pCE2 (lanes 1,2), psCE2 (lanes 3,4) and pC28-sCE2 (lanes 5,6) respectively are shown. The CE proteins migrated with an apparent molecular weight of 75 kDa whereas the fusion protein had a molecular weight of 100 kDa. As expected, CE2 mainly remained intracellular while sCE2 and C28-sCE2 were secreted by transfected COS-7 cells.

COS-7 cells were transfected with pCE2, psCE2 or pC28-sCE2 and expressed proteins in supernatant and cellular lysates were analysed by Western blotting, FACS analysis, esterase activity assay and cytotoxicity assays. To assess the size of the expressed proteins and determine the amount of secreted protein, SDS–PAGE was performed followed by Western blotting and detection with anti-myc antibody for sCE2 and C28-sCE2 or anti CE2 antibody for CE2 (Figure 2

). The CE2 protein appeared to remain intracellular since it was only detected in the cellular lysate (Figure 2, lane 1) of transfected COS-7 cells. Like CE2, the sCE2 monomers migrated with an apparent molecular weight of 75 kDa. As expected, the majority of sCE2 was detected in the supernatant of transfected COS-7 cells, proving that deletion of the C-terminal retention signal and fusing the Ig kappa leader, indeed directed the protein into the secretory pathway (Figure 2, lane 5). The C28-sCE2 fusion protein, with an apparent molecular weight of 100 kDa, was also found mainly in the supernatants of transfected COS-7 cells (Figure 2, lane 6).

**Figure 3:**
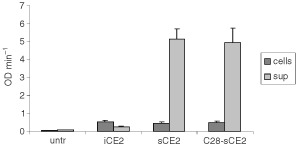
CE-activity in cellular lysates and supernatants of COS-7 transfected with pCE2, psCE2, pC28-sCE2. Cellular lysates or supernatants of transfected COS-7 cells were incubated with 100 mM pNpAc and conversion was measured during 10 min. sCE2 and C28-sCE2 show enzymatic activity and are efficiently secreted by transfected cells, because most of the enzyme activity is found in the supernatant.

Functional enzyme activity of CE2, sCE2 and C28-sCE2 was demonstrated by an esterase enzyme activity assay (Figure 3

). In cells transfected with pCE2 esterase activity remained intracellular, while in cells transfected with psCE2 or pC28-sCE2 almost all activity was detected in the culture medium. These results confirmed the results of the Western blotting experiments, since the relative amounts and the activities of CE2 proteins in cells and supernatants of transfected COS-7 cells were comparable.

**Figure 4:**
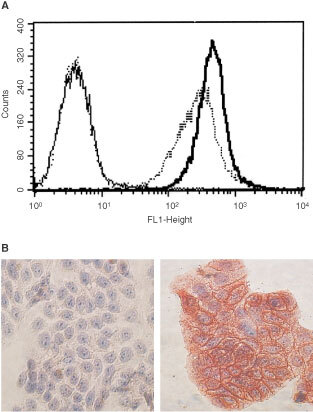
Binding of C28-sCE2 to the EpCAM expressing human colon cancer cell line SW1398. (**A**) FACS analysis of SW1398 cells that highly express EpCAM, with the supernatants of COS-7 cells transfected with psCE2 or pC28-sCE2. As a positive control the 323A3 antibody (bold line), directed to EpCAM was used. Binding was visualised with mouse anti-myc antibody and fluorescein-conjugated rabbit anti-mouse IgG. The fusion protein C28-sCE2 (dotted line) is able to bind to SW1398 cells, whereas sCE2 is overlapping the PBS control (solid line). (**B**) Cells were incubated for 24 h with supernatants of transfected COS-7 cells. Hereafter, cells were stained with anti-myc antibody to show binding of sCE2 or C28-sCE2 to EpCAM. Cells were counterstained with haematoxylin. Only cells incubated with C28-sCE2 (right) show binding of the fusion protein to the cellular membrane, whereas sCE2 incubation did not show bound protein (left).

Binding of the C28-sCE2 fusion protein to EpCAM was demonstrated by FACS analysis of EpCAM expressing SW1398 colon cancer cells incubated with transfected COS-7 supernatants (Figure 4A

), whereas sCE2 did not bind the EpCAM expressing cells. Thus, the C28 moiety of C28-sCE2 mediated EpCAM binding. Furthermore, SW1398 cells were plated and incubated with the transfected COS-7 supernatants for 24 h. Hereafter, cells were stained with anti-myc antibody to detect bound fusion protein. C28-sCE2 was detected at the membrane of SW1398 cells, shown in Figure 4B, whereas cells incubated with sCE2 were not stained.

### Prodrug activation and antiproliferative effects

Concentrated supernatants of COS-7 cells transfected with pCE2, psCE2 or pC28-sCE2 were analysed for CPT-11 conversion using HPLC. Supernatants were incubated with CPT-11 for 22 h at 37°C. It was found that both sCE2 and C28-sCE2, which were secreted in the culture medium of transfected cells, were able to activate the prodrug CPT-11, since the drug SN-38 was formed (data not shown).

**Figure 5:**
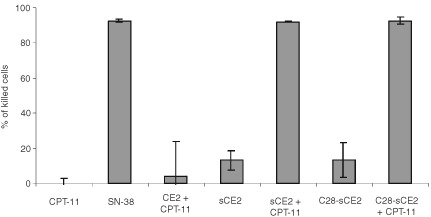
Cytotoxicity assay with the EpCAM expressing cell line SW1398 incubated with 1 μM CPT-11 and concentrated supernatants of COS-7 cells transfected with pCE2, psCE2 or pC28-sCE2 or with supernatants only. Results are shown as percentage of killed cells compared to untreated control cells, which were set to 0% kill. Incubation with sCE2 or C28-sCE2 supernatants and the non-toxic concentration of CPT-11 results in growth inhibition comparable to incubation with 1 μM SN-38, whereas incubation with CPT-11 or supernatant only is not toxic.

To show the effect of CPT-11 conversion into SN-38 by CE2, sCE2 and C28-sCE2 on the viability of colon cancer cells, the EpCAM-expressing colon carcinoma cell line SW1398 was incubated overnight with the concentrated supernatant of COS-7 cells transfected with pCE2, psCE2 or pC28-sCE2. After incubation, culture medium or a non-toxic concentration (1 μM) of CPT-11 was added. In Figure 5

it is shown that the supernatants of COS-7 cells transfected with sCE2 or C28-sCE2 render SW1398 cells susceptible to CPT-11. Incubation with supernatants of pCE2 transfected COS-7 cells and CPT-11, which do not secrete CE, or incubation with sCE2 or C28-sCE2 supernatant only, did not show augmented toxicity.

## DISCUSSION

CPT-11 is a prodrug for the treatment of colon cancer. One enzyme that converts CPT-11 into the toxic drug SN-38 is CE. By increasing the concentration of CE at the site of a tumour via a GDEPT approach, the conversion of CPT-11 to SN-38 will be enhanced at the site of the tumour, leading to tumour specific cytotoxicity. Human liver CE1 and rabbit CE have been employed in a GDEPT approach in combination with CPT-11. Although rabbit CE appeared to convert CPT-11 very effectively (Danks *et al*, 1999), an enzyme of human origin is preferred for *in vivo* applications to treat patients. Human CE1 showed a low conversion velocity and a low hydrolysis rate for CPT-11 in comparison with CE2 (Humerickhouse *et al*, 2000). Therefore, in this study we used the human liver CE2 enzyme to sensitise human tumour cells to CPT-11. Because current gene transfer technology does not allow expression of transgenes in all cells of a targeted tumour *in vivo*, a bystander effect is required, in order to achieve efficient tumour reduction. Extracellularly produced SN-38 should not only kill the tumour cells in which CE2 is formed, but also neighbouring tumour cells that do not express CE2. To investigate whether extracellular conversion of CPT-11 would lead to a larger bystander effect than intracellular conversion, we constructed a secreted form of CE2 (sCE2). Furthermore, we hypothesised that a fusion protein consisting of sCE2 fused to a tumour specific scFv antibody would be retained in the tumour thereby preventing leakage of the enzyme into the circulation and therefore further reducing unwanted side effects. An example of a tumour-associated antigen is EpCAM. This molecule is an attractive target for enzyme prodrug therapy, since it is highly expressed on the cell surface of most carcinomas, including colon tumours. Furthermore, EpCAM is highly expressed on distant metastasis (Litvinov *et al*, 1994). Therefore, we constructed a fully human fusion protein consisting of sCE2 fused to a human scFv antibody directed to EpCAM (C28-sCE2). Intratumoural expression of this protein in cancer patients is expected to be less immunogenic than expression of non-human fusion proteins.

The secreted and the targeted protein were detected in the supernatant of transfected COS-7 cells and the secreted proteins exhibited comparable enzymatic activities as determined by conversion of pNpAc. Comparing the secreted proteins to intracellular wildtype CE2, it was observed that transfecting COS-7 cells with pCE2 resulted in a much lower total amount of CE-activity than cells transfected with psCE2 or the fusion protein C28-sCE2. Whether this is due to a greater amount of protein or a higher enzyme activity of sCE2 when compared with CE is not clear. C28-sCE2 showed enzyme activity and specific binding to EpCAM expressing cells as determined by FACS analysis and immunohistochemistry on SW1398 cells, whereas sCE2 did not bind these cells. Furthermore, using HPLC analysis it was shown that the secreted as well as the targeted form of CE2 were able to efficiently convert CPT-11 into SN-38. Experiments with SW1398 colon carcinoma cells that were incubated with secreted or targeted protein and a non-toxic concentration of CPT-11 showed complete growth inhibition of these cells.

In conclusion, we constructed a secreted form of CE2 that was capable to convert the prodrug CPT-11, leading to enhanced toxicity of CPT-11 to colon cancer cells. This construct holds promise in GDEPT approaches since transduction of tumour cells with psCE2 will most likely result in high concentrations of sCE2 throughout the whole tumour. Therefore, CPT-11 will be converted to SN-38 very efficiently throughout the tumour, resulting in a larger bystander effect than intracellular conversion of CPT-11. The C28-sCE2 fusion protein is as active as sCE2, and therefore this construct is as useful as sCE2 for GDEPT, but the theoretical advantage of C28-sCE2 is that the targeting moiety will prevent leakage of the construct into the circulation. However, from this study it can not be concluded that C28-sCE2 will have this additional advantage as compared to sCE2. To prove this hypothesis, *in vivo* experiments are necessary in which sCE2 and C28-sCE2 are expressed in colon carcinoma xenografts followed by CPT-11 administration.

## References

[bib1] ArafatWGomez-NavarroJXiangJSiegalGPAlvarezRDCurielDT2000 Antineoplastic effect of anti-erbB-2 intrabody is not correlated with scFv affinity for its target Cancer Gene Ther 7 12501256 11023197 10.1038/sj.cgt.7700228

[bib2] AustinEAHuberBE1993 A first step in the development of gene therapy for colorectal carcinoma: cloning, sequencing, and expression of Escherichia coli cytosine deaminase Mol Pharmacol 43 380387 8450832

[bib3] ChanSGabraHHillFEvanGSikoraK1987 A novel tumour marker related to the c-myc oncogene product Mol Cell Probes 1 7382 3331171 10.1016/0890-8508(87)90008-9

[bib4] DanksMKMortonCLKrullEJCheshirePJRichmondLBNaeveCWPawlikCAHoughtonPJPotterPM1999 Comparison of activation of CPT-11 by rabbit and human carboxylesterases for use in enzyme/prodrug therapy Clin Cancer Res 5 917924 10213229

[bib5] De GraafMBovenEOosterhoffDVan der Meulen-MuilemanIHHulsGAGerritsenWRPinedoHM2002 A fully human anti-Ep-CAM scFv beta-glucuronidase fusion protein for selective chemotherapy with a glucuronide prodrug Br J Cancer 86 811819 11875747 10.1038/sj.bjc.6600143 PMC2375299

[bib6] EdwardsDPGrzybKTDresslerLGManselREZavaDTSledgeJrGWMcGuireWL1986 Monoclonal antibody identification and characterization of a Mr 43,000 membrane glycoprotein associated with human breast cancer Cancer Res 46 13061317 3510721

[bib7] GuptaEMickRRamirezJWangXLestingiTMVokesEERatainMJ1997 Pharmacokinetic and pharmacodynamic evaluation of the topoisomerase inhibitor irinotecan in cancer patients J Clin Oncol 15 15021510 9193346 10.1200/JCO.1997.15.4.1502

[bib8] HojringNSvensmarkO1977 Carboxylesterases of human brain extract. Purification and properties of a butyrylesterase Biochim Biophys Acta 481 500514 857894 10.1016/0005-2744(77)90283-2

[bib9] HoughtonJACheshirePJHallmanIIJDLutzLLuoXLiYHoughtonPJ1996 Evaluation of irinotecan in combination with 5-fluorouracil or etoposide in xenograft models of colon adenocarcinoma and rhabdomyosarcoma Clin Cancer Res 2 107118 9816097

[bib10] HoughtonPJCheshirePJHallmanIIJDLutzLFriedmanHSDanksMKHoughtonJA1995 Efficacy of topoisomerase I inhibitors, topotecan and irinotecan, administered at low dose levels in protracted schedules to mice bearing xenografts of human tumours Cancer Chemother Pharmacol 36 393403 7634381 10.1007/BF00686188

[bib11] HulsGAHeijnenIACuomoMEKoningsbergerJCWiegmanLBoelEvan der Vuurst de VriesARLoysonSAHelfrichWvan Berge HenegouwenGPvan MeijerMde KruifJLogtenbergT1999 A recombinant, fully human monoclonal antibody with antitumour activity constructed from phage-displayed antibody fragments Nat Biotechnol 17 276281 10096296 10.1038/7023

[bib12] HumerickhouseRLohrbachKLiLBosronWFDolanME2000 Characterization of CPT-11 hydrolysis by human liver carboxylesterase isoforms hCE-1 and hCE-2 Cancer Res 60 11891192 10728672

[bib13] KettermanAJBowlesMRPondSM1989 Purification and characterization of two human liver carboxylesterases Int J Biochem 21 13031312 2612723 10.1016/0020-711x(89)90149-3

[bib14] KojimaAHackettNRCrystalRG1998a Reversal of CPT-11 resistance of lung cancer cells by adenovirus-mediated gene transfer of the human carboxylesterase cDNA Cancer Res 58 43684374 9766666

[bib15] KojimaAHackettNROhwadaACrystalRG1998b In vivo human carboxylesterase cDNA gene transfer to activate the prodrug CPT-11 for local treatment of solid tumours J Clin Invest 101 17891796 9541511 10.1172/JCI119888 PMC508762

[bib16] LaemmliUK1970 Cleavage of structural proteins during the assembly of the head of bacteriophage T4 Nature 227 680685 5432063 10.1038/227680a0

[bib17] LitvinovSVVeldersMPBakkerHAFleurenGJWarnaarSO1994 Ep-CAM: a human epithelial antigen is a homophilic cell-cell adhesion molecule J Cell Biol 125 437446 8163559 10.1083/jcb.125.2.437 PMC2120036

[bib18] MooltenFL1986 Tumour chemosensitivity conferred by inserted herpes thymidine kinase genes: paradigm for a prospective cancer control strategy Cancer Res 46 52765281 3019523

[bib19] MungerJSShiGPMarkEAChinDTGerardCChapmanHA1991 A serine esterase released by human alveolar macrophages is closely related to liver microsomal carboxylesterases J Biol Chem 266 1883218838 1918003

[bib20] RivoryLPHaazMCCanalPLokiecFArmandJPRobertJ1997 Pharmacokinetic interrelationships of irinotecan (CPT-11) and its three major plasma metabolites in patients enrolled in phase I/II trials Clin Cancer Res 3 12611266 9815808

[bib21] SatohTHosokawaM1998 The mammalian carboxylesterases: from molecules to functions Annu Rev Pharmacol Toxicol 38 257288 9597156 10.1146/annurev.pharmtox.38.1.257

[bib22] SatohTHosokawaMAtsumiRSuzukiWHakusuiHNagaiE1994 Metabolic activation of CPT-11, 7-ethyl-10-[4-(1-piperidino)-1- piperidino]carbonyloxycamptothecin, a novel antitumour agent, by carboxylesterase Biol Pharm Bull 17 662664 7920428 10.1248/bpb.17.662

[bib23] SoneTWangCY1997 Microsomal amidases and carboxylesterases Compr Toxicol 3 265281

[bib24] ThompsonJZamboniWCCheshirePJLutzLLuoXLiYHoughtonJAStewartCFHoughtonPJ1997a Efficacy of systemic administration of irinotecan against neuroblastoma xenografts Clin Cancer Res 3 423431 9815701

[bib25] ThompsonJZamboniWCCheshirePJRichmondLLuoXHoughtonJAStewartCFHoughtonPJ1997b Efficacy of oral irinotecan against neuroblastoma xenografts Anticancer Drugs 8 313322 9180383 10.1097/00001813-199704000-00002

[bib26] TsujiTKanedaNKadoKYokokuraTYoshimotoTTsuruD1991 CPT-11 converting enzyme from rat serum: purification and some properties J Pharmacobiodyn 14 341349 1783980 10.1248/bpb1978.14.341

[bib27] WierdlMMortonCLWeeksJKDanksMKHarrisLCPotterPM2001 Sensitization of human tumour cells to CPT-11 via adenoviral-mediated delivery of a rabbit liver carboxylesterase Cancer Res 61 50785082 11431344

[bib28] ZhuWSongLZhangHMatoneyLLeCluyseEYanB2000 Dexamethasone differentially regulates expression of carboxylesterase genes in humans and rats Drug Metab Dispos 28 186191 10640517

